# How should we treat long-standing overt ventriculomegaly in adults (LOVA)? A retrospective cohort study

**DOI:** 10.1007/s10143-022-01812-5

**Published:** 2022-06-11

**Authors:** Conor S. Gillespie, George E. Richardson, Mohammad A. Mustafa, Daisy Evans, Alan M. George, Abdurrahman I. Islim, Conor Mallucci, Michael D. Jenkinson, Catherine J. McMahon

**Affiliations:** 1 Institute of Systems, Molecular and Integrative Biology, School of Medicine, University of Liverpool, Cedar House, Ashton Street, Liverpool, L69 3GE UK; 2 Department of Neurosurgery, The Walton Centre NHS Foundation Trust, Liverpool, UK; 3 School of Medicine, Keele University, Staffordshire, UK; 4 School of Medicine, University of Birmingham, Birmingham, UK; 5 Department of Neurosurgery, Alder Hey Children’s NHS Foundation Trust, Liverpool, UK

**Keywords:** LOVA, Long-standing overt ventriculomegaly in adults, Chronic hydrocephalus, Arrested hydrocephalus, Aqueduct stenosis, Hydrocephalus

## Abstract

Long-standing overt ventriculomegaly in adults (LOVA) is a heterogenous group of conditions with differing presentations. Few studies have evaluated success rates of available surgical treatments, or ascertained the natural history. There is a need to assess the efficacy of both endoscopic third ventriculostomy (ETV) and ventriculoperitoneal shunt (VPS) as first-line treatments. We conducted a retrospective, single-centre study of adults with LOVA at a tertiary neurosurgery centre in England, UK, aiming to identify presentation, management strategy, and outcome following treatment. A total of 127 patients were included (mean age 48.1 years, 61/127 male). Most patients were symptomatic (73.2%, *n* = 93/127, median symptom duration 10 months). The most common symptoms were gait ataxia, headache, and cognitive decline (52.8%, 50.4%, and 33.9%, respectively). Fourteen patients had papilloedema. Ninety-one patients (71.7%) underwent surgery (84 ETV, 7 VPS). Over a median follow-up of 33.0 months (interquartile range [IQR] 19.0–65.7), 82.4% had a clinical improvement after surgery, and 81.3% had radiological improvement. Clinical improvement rates were similar between ETV and VP shunt groups (82.1% vs 85.7%, *p* = 0.812). Surgical complication rates were significantly lower in the ETV group than the VP shunt group (4.8% vs 42.9%, *p* < 0.001). Of the patients treated surgically, 20 (22.0%) underwent further surgery, with 14 patients improving. This study demonstrates the efficacy of ETV as a first-line treatment for LOVA.

**Supplementary Information:**

The online version contains supplementary material available at 10.1007/s10143-022-01812-5.

## Introduction

Long-standing overt ventriculomegaly in adults (LOVA) describes a heterogenous group of conditions, consisting of chronic ventriculomegaly in the absence of raised intracranial pressure (ICP) [16]. LOVA encapsulates conditions including arrested hydrocephalus, chronic congenital hydrocephalus, aqueduct stenosis with hydrocephalus, and syndrome of hydrocephalus in young and middle aged adults [6]. LOVA presents in adulthood with varied symptoms, ranging from being discovered incidentally on routine imaging, to acute symptoms suggestive of raised ICP [19, 22]. LOVA is being increasingly recognised as a distinct entity in clinical practice, and how to manage the condition and best ascertain its natural history is an important clinical problem [7].

The optimal treatment for symptomatic LOVA is yet to be defined. Previous low-powered studies [19, 22] have suggested that both endoscopic third ventriculostomy (ETV) and ventriculoperitoneal shunt (VPS) are effective, with proponents of both treatments [2, 17]. There is a need to identify the best treatment for newly diagnosed or symptomatic LOVA, and to identify the long-term success rates of both interventions.

## Materials and methods

### Study design, setting, and participants

We conducted a single-centre, retrospective cohort study of all adults ≥18 years newly diagnosed with LOVA between 1st January 2003 and 10th October 2020. The study was approved by the hospital audit committee. Patients with hydrocephalus due to a secondary cause (e.g. tumour, intraventricular haemorrhage at birth, structural or developmental abnormality) were excluded. The study setting was a tertiary neuroscience centre in England, UK, with a catchment area of 3.5 million people. Patients were identified either incidentally or through symptomatic presentation. A radiological picture archiving and communications systems (PACS) scan database was searched for the terms ‘LOVA’, ‘Arrested hydrocephalus’, ‘Aqueduct stenosis’, and ‘Chronic hydrocephalus’ reported by radiologists in CT and MRI head scans conducted during the study period, cross-referenced against all patients with the operation code for endoscopic third ventriculostomy during the study period, and records screened for eligibility. Patients were diagnosed by a board-certified neurosurgeon (CJM, CM, or MDJ) with expertise in CSF disorders. They defined LOVA as satisfying at least two of the original diagnostic criteria defined by Oi et al.: (1) overt ventriculomegaly involving the lateral and third ventricles, (2) clinical symptoms with or without macrocephaly, and (3) evidence of expanded or destroyed sella turcica as evidence of long-standing ventriculomegaly [16].

### Baseline characteristics

Baseline characteristics included date and age at diagnosis (with date being defined as the date the patient had imaging suggestive of LOVA), diagnosing clinician, use of ICP monitoring, head circumference when recorded, clinical symptoms and nature of presentation, examination findings including papilloedema if present, and LOVA cluster class as defined by Craven et al. [7]. Radiological characteristics of LOVA included presence of triventriculomegaly, aqueduct stenosis, frontal and occipital horn width, and Evans’ index (ratio of maximum width of frontal horns to maximum internal skull diameter). Inter- and intra-rater reliability of Evans’ index was assessed on all patients by 2 observers independently (GER and MAM) using the intraclass correlation coefficient (ICC) [3].

### Management data

Management decision at diagnosis was stratified into active monitoring or intervention (surgery). For patients who underwent surgery, the type of surgery (ETV or VP shunt) and surgical morbidity were recorded. The primary outcome was patient-reported clinical improvement after surgical intervention (defined by the patient as a reduction or resolution of symptoms). For patients with LOVA discovered incidentally, the primary outcome was development of symptoms during the follow-up period. Secondary outcomes included radiological improvement after surgical intervention (defined as a decrease in ventricular size) and ETV success grade according to three expert defined definitions (Ibanez-Botella, Oi, and Jenkinson) [9, 16, 20]. All definitions include evidence of clinical improvement and radiological stability, with one including a reduction of ventricular size in success criteria. Further interventions (repeat surgery) and WHO performance status [21] at last follow-up were recorded.

### Statistical analysis

Data analysis was conducted using R V4.0.2, and figures displayed using RStudio (ggplot2 and blandr packages). Continuous variables were subject to a Kolmogorov-Smirnov test of normality—normally distributed variables are presented using mean and standard deviation (SD), and skewed variables using median and interquartile range (IQR). The chi-squared and Fisher’s exact test were used to compare success and complication rates between the two treatment groups, with a *p* value ≤ 0.05 considered statistically significant.

## Results

### Baseline, clinical, and radiological features

**Fig. 1:**
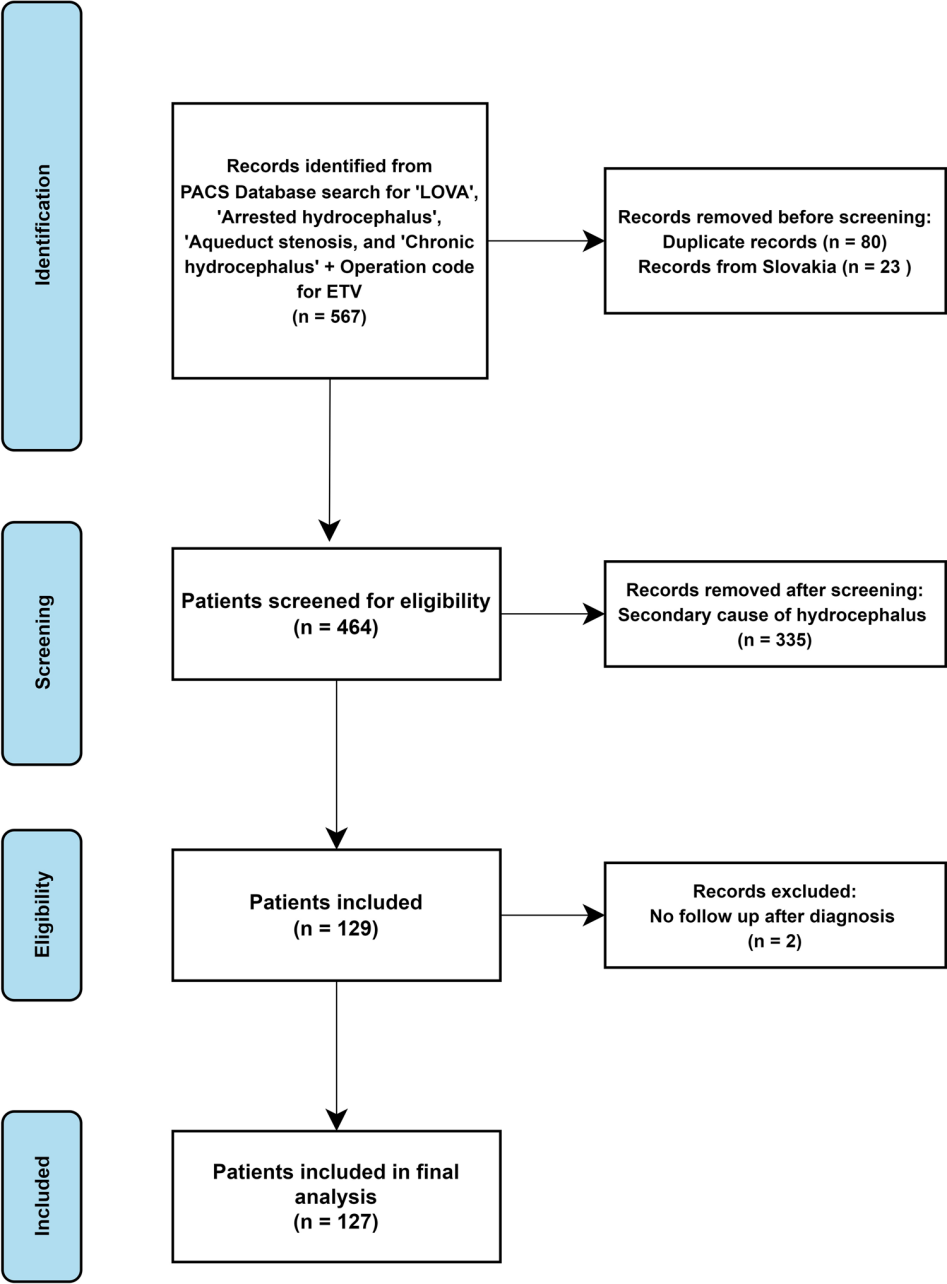
Flow chart showing patient identification, screening, and inclusion

**Fig. 2:**
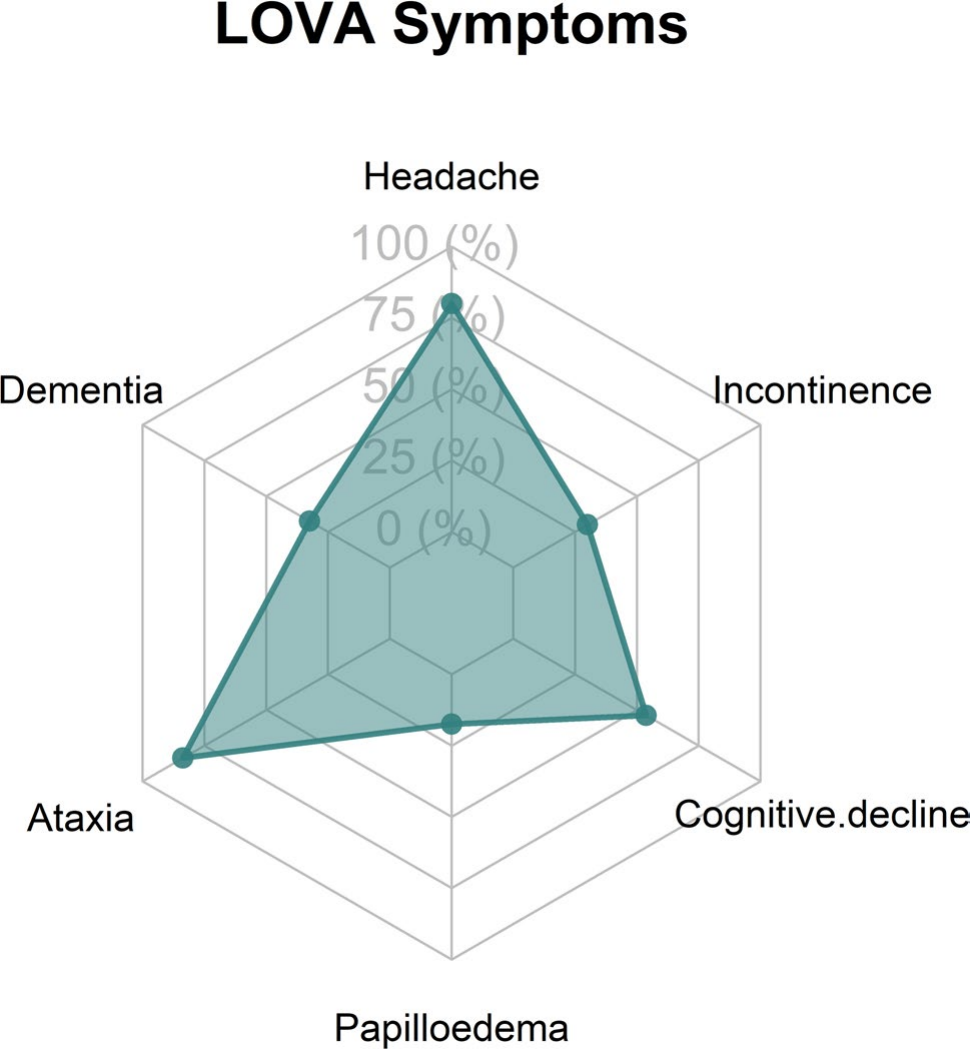
Radar plot demonstrating presenting symptom frequency of the cohort

Five hundred sixty-seven potentially eligible patients were identified using the database search. We excluded 80 duplicate records (patients identified from both CT and MRI head scans), 23 patients from Slovakia with no LOVA diagnosis (as the search term ‘LOVA’ appears in ‘Slovakia’), and 335 patients due to secondary cause for their hydrocephalus, leaving 129 patients (Fig. 1). After excluding two patients without any follow-up after diagnosis, 127 patients were included. The baseline, clinical, and radiological characteristics are outlined in Table 1. The mean age at diagnosis was 48.1 years of age (range 21–81). Forty-eight percent were male (*n* = 61/127). Most patients were symptomatic (73.2%, *n* = 93/127). The 34 patients discovered incidentally had MRI and CT head scans performed for mild head injury (*n* = 6), syncope (*n* = 4), migraine (*n* = 4), seizures (*n* = 4), stroke (*n* = 4), neck pain (*n* = 3), back pain (*n* = 3), and other reasons (*n* = 6). For symptomatic patients, the median duration of symptoms before diagnosis was 10 months (IQR 5–27 months). The most common symptoms were gait ataxia (52.8%, *n* = 67), headache (50.4%, *n* = 64), and cognitive decline (33.9%, *n* = 43) (Fig. 2). On examination, all 20 patients that had a head circumference measurement available had macrocephaly (>98th percentile). All patients met at least two of the LOVA diagnostic criteria defined by Oi et al. [16]. Fourteen patients (6.3%) had evidence of papilloedema on examination.

**Table 1:** Baseline characteristics

Baseline characteristics	Value
Total patients	127
Mean age at diagnosis (SD)	48.1 (15.6)
Female, *n* (%)	66 (52.0)
Symptomatic (%)	93 (73.2)
Median symptom duration in months (IQR)	10 (5–27)
Symptoms	Frequency (%)
Headache	64 (50.4)
Gait ataxia	67 (52.8)
Cognitive impairment/decline	43 (33.9)
Urinary incontinence	24 (18.9)
Dementia	26 (20.5)
Papilloedema	14 (11.0)
Parkinsonism	8 (6.3)
Psychiatric disturbance	4 (3.1)
Cluster class	Frequency (%)
1	41 (32.3)
2	28 (22.0)
3	25 (19.7)
4	33 (26.0)
5	0 (0)
Radiological features	Frequency (%)
Triventriculomegaly	124 (97.6)
Lateral horn enlargement	125 (98.4)
Aqueduct stenosis	111 (87.4)
Panventriculomegaly	12 (9.4)
Sellar enlargement/destruction	66 (52.0)
Mean Evans index (range)	0.45 (0.31–0.71)
Treatment strategies	Frequency (%)
Conservative	36 (28.3)
ETV	84 (66.1)
VPS	7 (5.5)
Median follow-up in months (IQR)	33.0 (19.0–65.7)

*SD* standard deviation, *IQR* interquartile range, *ETV* endoscopic third ventriculostomy, *VPS* ventriculoperitoneal shunt

97.6% of patients had evidence of triventriculomegaly on neuroimaging (*n* = 124), 98.4% lateral horn enlargement (*n* = 125), 87.4% aqueduct stenosis (*n* = 111), 18.9% fourth ventricle enlargement (*n* = 24), and 47.2% had sellar destruction or enlargement (*n* = 47.2). All patients had an Evans’ index >0.3, and 79.5% (*n* = 101/127) an index >0.4 (mean 0.45, SD 0.07). The ICC showed good reliability between measurements (mean 0.94, 95% CI 0.91–0.96) (Electronic supplementary material).

### Management strategy at diagnosis

Forty-two patients (33.1%) were managed conservatively at diagnosis. Of the conservatively managed group, over a mean follow-up of 36.0 months (SD 28.3 months) seven developed symptoms (16.7%) and were managed with surgery in six cases. In total, 91 (71.7%) were managed with surgery (median time to surgery from diagnosis 4.1 months, IQR 1.4–8.4 months). The median follow-up of the cohort was 33.0 months (IQR 19.0–65.7).

### Surgical details

**Fig. 3:**
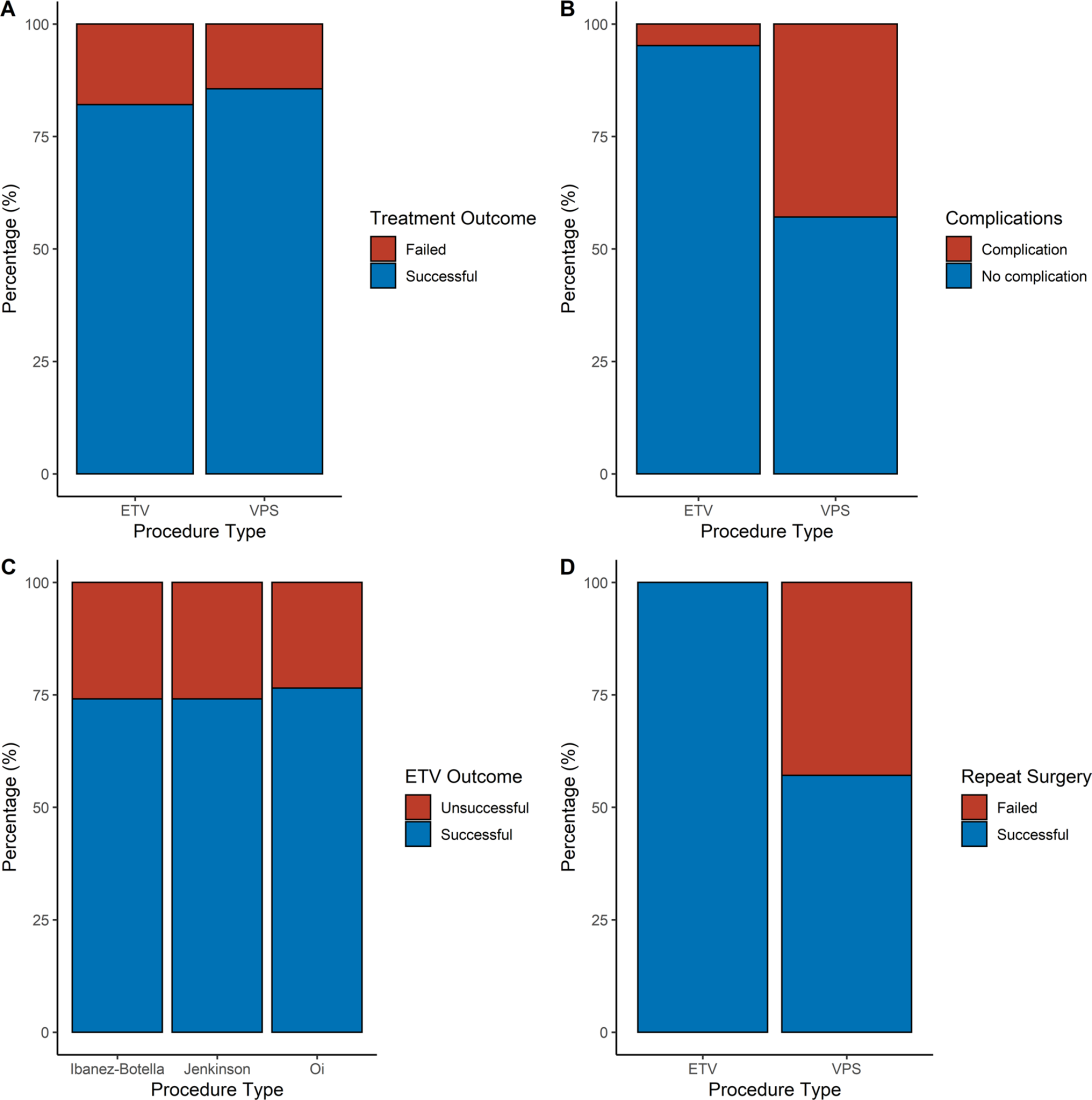
Stacked bar charts demonstrating **a** success rates of two procedures, **b** difference in complication rates between ETV and VP shunt groups, **c** success rates according to expert defined definitions, and **d** success of repeat surgery

In total, 92.3% were ETVs (*n* = 84), and 7.7% VPS (*n* = 7). VPS was selected as first-line treatment in four patients who presented with predominantly NPH symptoms (ataxia, cognitive decline and urinary incontinence, with symptom improvement following lumbar drain procedures), and in three patients with unfavourable anatomy (two atypical ventricular dilatation and one pre-pontine cistern effacement), indicating reduced probability of ETV success. VPS had a significantly higher frequency of surgical complications (42.9% vs 4.8%, *p* < 0.001) (Fig. 3). In the VPS group, there was one patient with an infected shunt, one patient with a wound and CSF leak, and one patient who developed abdominal sepsis from the operation, requiring an emergency laparotomy. In the ETV group, two patients had wound infections that resolved with antibiotics, one patient developed hospital acquired pneumonia, which resolved after being treated with intravenous antibiotics, and one patient had a CSF leak, requiring an external ventricular drain. Of the complications, none persisted within 30 days or caused mortality—one VP shunt was replaced after a wound leak, the shunt removed, and the infection treated with intrathecal antibiotics via an external ventricular drain.

### Surgical success rates and follow-up

82.4% of patients experienced a clinical improvement after surgery (Table 2). This was a permanent improvement in 82.9% (*n* = 63), and transient in 17.1% (*n* = 13, symptom benefit lasting for a mean of 23.7 months, with symptoms returning to pre-surgery levels). There was no difference in the success rates for both ETV and VP shunt groups (82.1% vs 85.7%, *p* = 0.812) (Fig. 3).

**Table 2:** Treatment outcomes for patients undergoing surgical intervention

**Treatment outcomes**	**Frequency (%)**	***p* value**
Clinical improvement (overall)	75 (82.4)	–
ETV	69 (82.1)	0.812
VPS	6 (85.7)	
Radiological improvement (overall)	74 (81.3)	–
ETV	68 (81.0)	0.756
VPS	6 (85.7)	
Symptom resolution at last follow-up (overall)	66 (72.5)	–
ETV	61 (72.6)	0.946
VPS	5 (71.4)	
Re-operation	20 (22.0)	–
ETV	6 (30.0)	
VPS	14 (70.0)	
Successful (overall)	14 (70)	–
ETV	6 (100)	0.055
VPS	8 (57.1)	
ETV success rates		–
Oi	65 (76.5)	
Jenkinson	63 (74.1)	
Ibáñez-Botella	63 (74.1)	

*ETV* endoscopic third ventriculostomy, *VPS* ventriculoperitoneal shunt

ETV resulted in a clinical improvement and reduction in ventricular size in 81.3% of cases, and clinical improvement with no reduction in ventricular size in 82.1% of cases (Fig. 3). At 1-year post follow-up, 75.0% (*n* = 63) had symptom resolution after an ETV, and 72.6% (*n* = 61) of ETV treated patients had symptom resolution at last follow-up.

Twenty-two patients (24.2%) had symptom recurrence after surgery. Twenty of these patients went onto have repeat surgery, with fourteen patients being treated with VP shunt (two replacing a previous VPS, twelve after unsuccessful ETV), and six with an ETV (repeat ETV in four patients, after failed VPS in two patients). Of these, fifteen improved (75.0%). The improvement seen after repeat surgery was similar in the ETV group compared to the VPS group (100% vs 57.1%, Fischer’s exact test = 0.055). Two patients had a third surgery—one patient had two unsuccessful VP shunts that were later converted to a ventriculoatrial (VA) shunt, and one patient had a VP shunt that required revision due to valve blockage, followed by symptom recurrence, then an ETV, with subsequent symptom resolution. No patients were lost to follow-up, and 41.7% (*n* = 74) were discharged from routine monitoring by the end of the study period.

## Discussion

In this retrospective cohort study of 127 adults with LOVA, 73.2% of patients were symptomatic. Over a median follow-up of 33 months, 82.4% of LOVA patients had symptom resolution after ETV or VPS, with no difference in clinical improvement between the two groups—this improvement was maintained at last follow-up. The surgical complication rate was significantly lower in patients treated with ETV, although almost 20% of patients undergoing surgery required a further operation for symptom recurrence.

The heterogenous symptoms and presentations associated with LOVA are in keeping with previously reported literature. One possible explanation for gait ataxia being the most common symptom is possible overlap with normal pressure hydrocephalus (NPH) [12]—Craven et al. noted a cluster of patients with LOVA that ‘mimic’ NPH clinically, but have imaging findings more characteristic of LOVA. The potential mechanism for ETV being successful in LOVA has been investigated previously and includes restoration of normal CSF flow and reduced pressure [1, 12]. The underlying pathophysiology behind LOVA has been described, with patients developing hydrocephalus that does not manifest clinically in infancy and young adulthood, before developing symptoms in later decades through an unknown mechanism [16, 18]. Several theories have been proposed, including that patients with LOVA have neither a fixed progressive or arrested hydrocephalus, but a transient state that shifts depending on certain time periods, leading to eventual failure of compensational CSF dynamic factors.

Previous studies of hydrocephalus due to secondary causes (space occupying lesions, infections, and infant hydrocephalus) have demonstrated ETV to have a lower complication rate, but similar success rates overall to VPS [4, 13, 15], in keeping with the results of our study. The study supports the observation that both treatments may be equally effective when used for symptomatic LOVA, but that ETV has lower morbidity. There may also be a role for repeat ETV, given the high success rate in leading to symptom resolution when used as repeat surgery. The reduced effectiveness rates also suggest that VPS after a failure of ETV as a first-line treatment may not be as successful.

The natural history and symptom presentation are variable, in keeping with a recent cluster analysis, which grouped LOVA into 5 clinical categories [7]. Cluster 1 represents those with incidental ventriculomegaly; cluster 2 being a highly symptomatic group who present acutely; cluster 3 a younger group with predominantly headaches as presenting symptom; cluster 4 an older group with symptoms similar to NPH; and cluster 5 a group with panventriculomegaly. The relatively equal cluster distributions in our cohort support this analysis, although no patients were recorded in group 5, a mixed responsiveness group.

Our success rates for ETV are similar to that reported in previous studies, which identified ETV to be between 75 and 84% effective when used for LOVA [7, 9, 12]. Many authors have postulated ETV to be the most optimal treatment for symptomatic LOVA based on these results. The success rates, as defined by three separate authors, are in keeping with published studies analysing ETV efficacy using clinical symptom improvement as the sole outcome measure [9, 10, 20], and provide further evidence that ETV leads to symptom resolution and ‘success’ between 75 and 85% of cases [2, 9].

Conservative management was employed in 42 patients, with 7 (16.7%) later going on to develop clinical symptoms. Non-surgical management strategies have not been thoroughly explored for LOVA. In a 2019 study of patients with adult hydrocephalus, 3 patients in the asymptomatic/mild symptom group were successfully treated with acetazolamide 250 mg twice daily, which resulted in resolution of clinical symptoms [7]. The use of acetazolamide is not employed routinely at our institution, however could be explored in future work to determine its utility for mildly symptomatic patients, or to prevent emergence of clinical symptoms in patients who are asymptomatic. This has been previously explored in patients with NPH [11], which may indicate potential for patients with LOVA who present with predominantly NPH-like symptoms.

### Study limitations

Our study is limited by its single-centre, retrospective nature, and by this virtue not all patients had available data, most notably head circumference measurements. In addition, not all patients had ICP monitoring to support the clinical and radiological diagnosis. Furthermore, LOVA is an increasingly utilised diagnosis and may therefore not have been described previously, with most cases being identified between 2010 and 2020 in our series, despite the enhanced search period.

Secondly, the decision to treat was based on clinical consensus and pragmatic benefit for the patient—this may not represent practice across the UK and internationally. It is unclear how management preferences for intervention impacted the results. Third, we did not assess or compare neuropsychological outcomes of patients, and between patient groups, however ETV has been suggested to lead to significant improvements in memory, attention, and concentration in just under half of patients treated in other studies [5, 8].

Finally, while the number of ETVs performed in this series is one of the largest in the existing literature, the limited number of VP shunts performed (*n* = 7) limits the generalisability of the findings for this treatment. The authors welcome others to share their experiences regarding this treatment, which is often included as part of mixed case series, thus precluding comparative analysis [14].

Our findings have several implications for practice. They indicate that ETV efficacy is similar to VPS in a direct comparison between the two groups, however the reduced complication rate in ETV may be beneficial to patients and treating clinicians. The study also provides evidence of the natural history and presentation of LOVA, supporting previous findings of the heterogenous presentation. The fact that 16.6% of asymptomatic patients managed with routine monitoring will also develop symptoms and require surgery has not been explored in previous studies.

## Conclusions

LOVA is a less commonly reported, but increasing clinical problem, with no clear consensus on optimal management strategy for symptomatic patients. ETV and VPS were equally effective in contributing to symptom resolution in our cohort, with ETV having a lower complication rate. This confirms the efficacy of ETV as a first-line treatment for symptomatic LOVA, with further studies required to identify and compare ETV further to VPS.

## Supplementary Information

Below is the link to the electronic supplementary material.

Supplementary file1 (DOCX 187 KB)

## Data Availability

Anonymized data are available (upon reasonable request) from the corresponding author.
