# Supplementary material for: How should we treat long-standing overt ventriculomegaly in adults (LOVA)? A retrospective cohort study
Source: Neurosurg Rev. 2022 Jun 11;45(5):3193–200. doi: 10.1007/s10143-022-01812-5 (PMC9492616; doi:10.1007/s10143-022-01812-5)
Supplement: Supplementary file 1 — Supplementary file1 (DOCX 187 KB) [file 10143_2022_1812_MOESM1_ESM.docx]

**Online only supplementary material**

Title: Long standing overt ventriculomegaly in adults (LOVA)- which treatment should be offered first?

Neurosurgical review

Authors: Gillespie CS, Richardson GE, Mustafa MA, Evans D, George AM, Islim AI, Mallucci C, Jenkinson MD, McMahon CJ.

Corresponding author email address: [M.A.Mustafa@liverpool.ac.uk](mailto:M.A.Mustafa@liverpool.ac.uk)


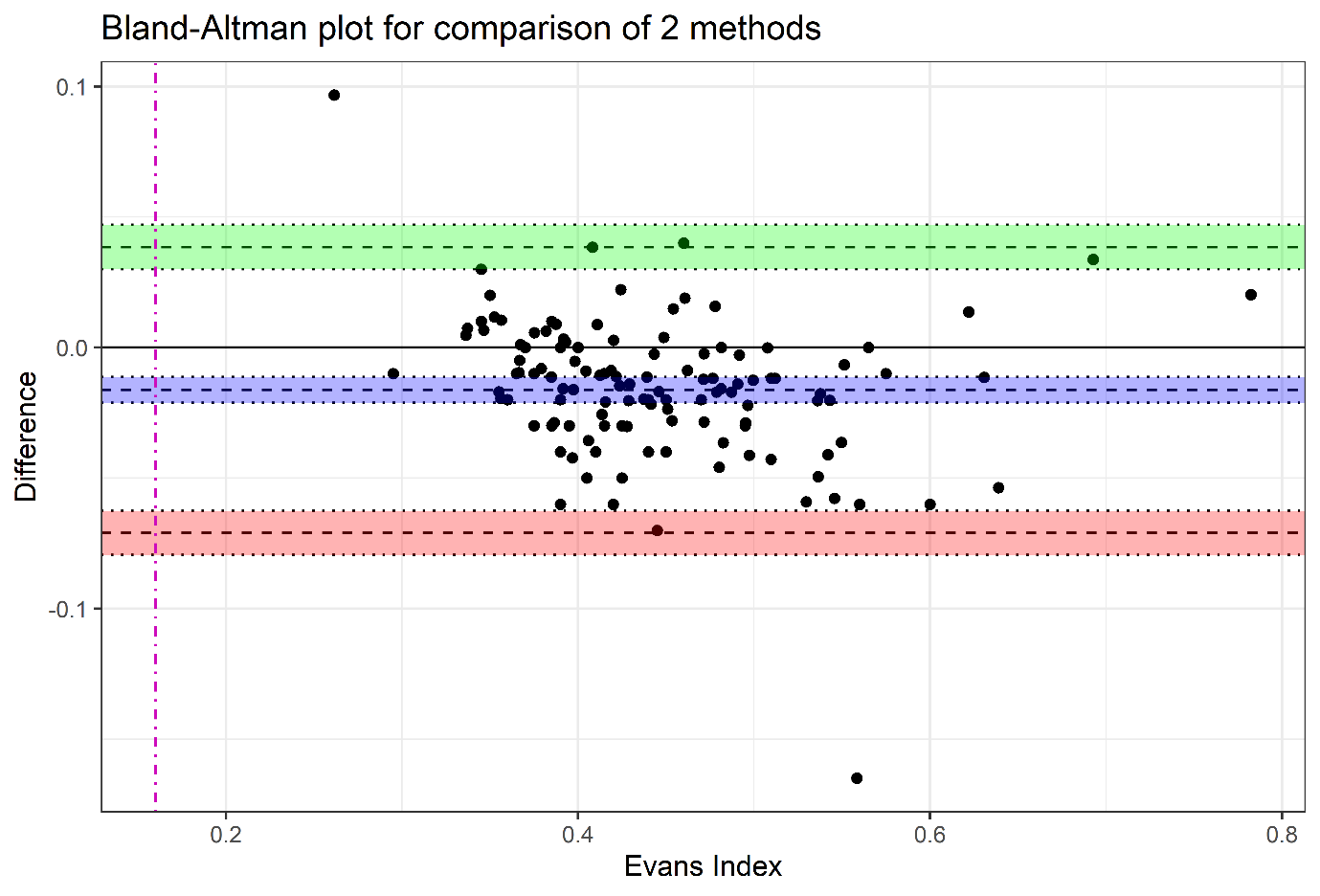


Supplementary Figure S1: Bland-Altman plot showing agreement between independent Evans Index measurements by authors GER and MAM.

| **Meningioma volume Intraclass correlation coefficient (95% CI)** | |
| --- | --- |
| Inter-rater variability^A^ | Intra-rater variability^B^ |
| 0.94 (95% CI 0.91-0.96) | 0.96 (95% CI 0.94-0.97) |

^A^Set to two-way mixed

^B^Set to one-way random

Supplementary table S1: Intraclass correlation coefficient (ICC) with a 95% Confidence interval displaying inter-rater variability and intra-rater variability between two independent authors, GER and MAM.
